# Clinical Outcomes and Factors Associated With Length of Stay Among Burn Patients With Pre‐Existing Neurological Disorders: A Retrospective Cohort Study

**DOI:** 10.1002/hsr2.72915

**Published:** 2026-07-28

**Authors:** Siamak Rimaz, Hossein Khoshrang, Mohammadreza Mobayen, Mona Alipour, Mohaya Farzin

**Affiliations:** ^1^ Anesthesiology Research Center, Department of Anesthesiology, Alzahra Hospital Guilan University of Medical Sciences Rasht Iran; ^2^ Burn and Regenerative Medicine Research Center Guilan University of Medical Sciences Rasht Iran; ^3^ Razi Clinical Research Development Unit Guilan University of Medical Sciences Rasht Iran

**Keywords:** burns, comorbidity, length of stay, nervous system diseases, operative, surgical procedures

## Abstract

**Background:**

Burn injuries can lead to significant systemic complications. Patients with pre‐existing neurological disorders may present additional clinical complexity during burn management; however, outcomes and associated factors in this subgroup are not well described.

**Objective:**

To describe clinical outcomes and identify factors associated with length of hospital stay among burn patients with pre‐existing neurological disorders admitted to a tertiary care center.

**Methods:**

This retrospective cohort study included 67 burn patients with documented pre‐existing neurological disorders identified from hospital records (2022–2024). Data collected included demographics, burn characteristics (total body surface area [TBSA], depth), number of surgical interventions, ICU admission, ventilator use, length of hospital stay, and discharge outcome. Analyses included descriptive statistics, sex‐based group comparisons, correlation analysis, and negative binomial regression to assess factors associated with hospital stay (SPSS v26; *p* < 0.05).

**Results:**

The mean age was 51.5 ± 21.7 years, and 52.2% of patients were female. Mean TBSA was 15.5% ± 20.5%. Mean length of stay was 3.5 ± 5.2 days (median 1 day). Most patients were discharged with partial recovery (74.6%); mortality was 4.5% (95% CI: 0.9%–12.5%). No significant sex differences were observed in key clinical variables. Length of stay was strongly correlated with the number of surgical interventions (*r* = 0.796, *p* < 0.001). In multivariable negative binomial regression, the number of operations was significantly associated with hospital length of stay. Because this variable accumulates during hospitalization, the observed association should be interpreted as descriptive rather than causal.

**Conclusion:**

Among burn patients with pre‐existing neurological disorders, a greater surgical burden was strongly associated with prolonged hospitalization. These findings support early identification of neurological comorbidity and coordinated multidisciplinary care to manage resource use and optimize outcomes in this high‐risk subgroup. Due to the absence of a comparator group and the retrospective design, causal inferences cannot be drawn.

## 1 Introduction

Burn injuries remain a major global health problem, with an estimated 11 million medically attended cases and more than 180,000 deaths each year [1]. Clinical severity is primarily determined by burn size and depth, but outcomes are also influenced by burn location, patient age, and underlying systemic comorbidities [1, 2]. Beyond the acute insult, burns can have long‐term consequences, including prolonged hospitalization and a range of secondary morbidities [3]. Burn mechanisms are diverse (thermal, electrical, chemical, friction, and radiation), and injuries are commonly classified by depth as superficial, superficial partial‐thickness, deep partial‐thickness, and full‐thickness burns [2]. Importantly, burn injury is not confined to the skin; it triggers sustained inflammatory and hypermetabolic responses that can impair multiple organ systems [2, 3]. Reported mortality rates vary (approximately 2.3%–3.6%), with sepsis and multiple organ failure among the most frequent causes of poor prognosis and death [2, 4]. The pathophysiology involves widespread inflammatory activation and tissue responses that promote metabolic derangements, systemic inflammatory response, energy depletion, and subsequent organ dysfunction [4].

One area that remains comparatively underexplored is the interaction between burn‐related systemic inflammation and the central nervous system (CNS). Proinflammatory mediators released by resident and circulating immune cells (e.g., macrophages and T cells) can disseminate across organs and amplify stress and immune signaling [3]. Patients with pre‐existing neurological disorders may represent a particularly vulnerable subgroup among burn patients [5]. Conditions such as stroke, epilepsy, dementia, Parkinson's disease, spinal cord injury, and neuromuscular disorders can influence mobility, cognition, sensory perception, and functional independence [6]. These impairments may increase susceptibility to burn injury, complicate acute management, delay rehabilitation, and potentially affect outcomes, including hospitalization and resource utilization. Despite these plausible clinical challenges, studies specifically evaluating burn outcomes among patients with neurological comorbidities remain limited [7]. Few studies have specifically characterized burn outcomes in patients with neurological comorbidities, and most focus on general comorbidity indices rather than neurological subgroups. Therefore, understanding clinical characteristics and factors associated with hospitalization in this subgroup may help improve multidisciplinary burn care strategies [3].

Given the scarcity of data, this study aims to provide a descriptive profile of this subgroup rather than establish causal relationships. Against this background, the present study aimed to describe outcomes among burn patients with pre‐existing neurological conditions admitted to a tertiary educational hospital in Rasht, Iran—a major referral center for burn care in northern Iran serving both urban and rural populations. We evaluated key clinical indicators, including TBSA, length of hospital stay, ICU admission, surgical burden, and mortality in this neurological comorbidity subgroup.

## 2 Materials and Methods

### 2.1 Study Design and Study Population

This retrospective cohort study was conducted at the Burn Center of Poursina Hospital, Rasht, Iran. Medical records of burn patients (2022–2024) were retrospectively reviewed. The study included patients with documented pre‐existing neurological disorders who met the study eligibility criteria.

Patients with incomplete medical records were excluded from the study. Only patients with a documented neurological disorder before the burn injury were included in the final analysis. A total of 67 eligible patients fulfilled the inclusion criteria and were included in the statistical analyses.

Inclusion criteria were: (1) confirmed burn injury requiring hospital admission and (2) verified pre‐existing neurological disorder as defined above. Patients with incomplete documentation for key variables (e.g., TBSA, length of stay, or number of operations) were excluded.

The attending burn surgeon classified burn depth as superficial partial‐thickness, deep partial‐thickness, or full‐thickness according to clinical examination.

Discharge status was determined from the physician's discharge summary documented in the medical record.

### 2.2 Data Collection

Clinical and demographic information was extracted retrospectively from hospital medical records using a structured data collection form developed for the study. Variables collected included age, sex, marital status, occupation, total body surface area burned (TBSA), burn depth, cause of burn, anatomical burn location, duration of hospitalization, intensive care unit admission, need for mechanical ventilation, number of surgical procedures, discharge outcome, and mortality.

Pre‐existing neurological disorders were identified from the documented medical history recorded in the patients' medical records. Neurological disorders were subsequently classified into three categories according to the principal anatomical site involved: central nervous system disorders, spinal cord disorders, and peripheral nervous system/neuromuscular disorders. Representative diagnoses included cerebrovascular disease, dementia, epilepsy, Parkinson's disease, neurodevelopmental disorders, spinal cord injury, peripheral neuropathy, and neuromuscular disorders. When multiple neurological diagnoses were present, patients were classified according to the principal neurological diagnosis documented in the medical record.

Because this was a retrospective medical record review, standardized assessments of neurological severity and baseline functional status were not consistently available and therefore were not included in the analysis.

Medical records were reviewed by two trained investigators using a standardized data extraction form. Uncertain neurological diagnoses were discussed with the senior burn surgeon until consensus was reached. Random samples of extracted records were independently checked for consistency before analysis.

Preliminary analyses demonstrated substantial overdispersion (variance exceeding the mean); therefore, negative binomial regression was selected instead of Poisson regression.

### 2.3 Statistical Analysis

Statistical analyses were performed using IBM SPSS Statistics version 26.0 (IBM Corp., Armonk, NY, USA). Continuous variables were assessed for normality using the Kolmogorov–Smirnov test and are presented as mean ± standard deviation (SD) or median (interquartile range [IQR]), as appropriate. Categorical variables are presented as frequencies and percentages.

Comparisons between male and female patients were performed using the independent‐samples *t*‐test for continuous variables and the chi‐square or Fisher's exact test for categorical variables, as appropriate. Correlations between continuous variables were assessed using Pearson's correlation coefficient.

Hospital length of stay was defined as the number of calendar days between hospital admission and discharge. Patients discharged on the same day were assigned a hospital length of stay of 0 days in the hospital registry.

Because hospital length of stay represented count data and demonstrated overdispersion (variance greater than the mean), a multivariable negative binomial regression with a log‐link function was used to evaluate factors associated with hospital length of stay. Age, total body surface area (TBSA), and the number of operations were entered simultaneously into the multivariable model. Results are presented as incidence rate ratios (IRRs) with corresponding 95% confidence intervals (CIs) and two‐sided *p* values.

Missing data were assessed before statistical analysis. Complete data were available for all variables included in the descriptive and multivariable analyses; therefore, no imputation procedures were required, and all 67 eligible patients were included in the final analyses.

A two‐sided *p* value of less than 0.05 was considered statistically significant.

## 3 Results

**Figure 1:**
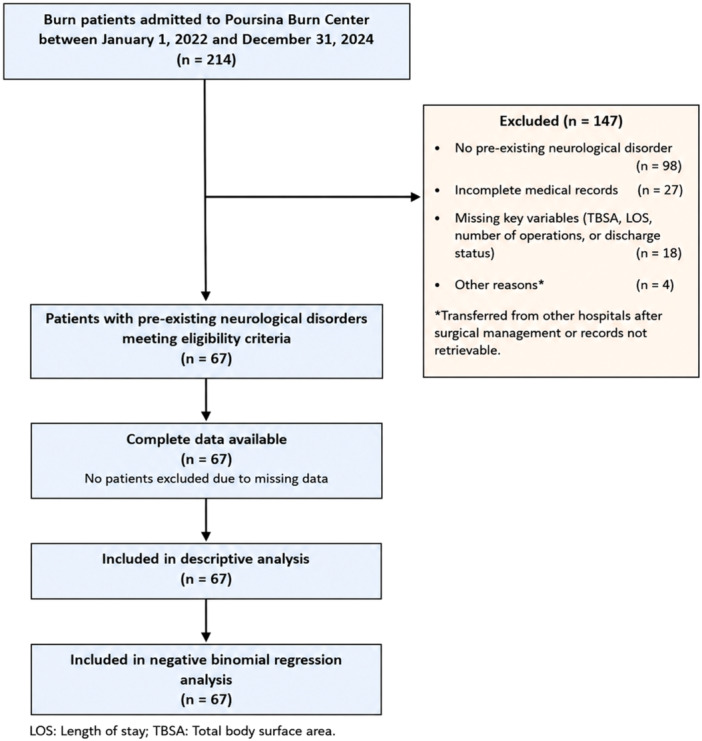
Flow diagram of patient selection and inclusion in the study. The total number of burn admissions during the study period was 214. Of these, 147 patients were excluded for the reasons shown above. Sixty‐seven patients met the inclusion criteria and had complete data for all study variables; therefore, all 67 patients were included in the final analyses.

A total of 67 burn patients with documented pre‐existing neurological disorders met the study eligibility criteria and were included in the final analysis (Figure 1). Complete data were available for all variables included in the descriptive and multivariable analyses; therefore, no observations were excluded because of missing data (Table 1).

**Table 1:** Baseline demographic and clinical characteristics of burn patients with pre‐existing neurological disorders (*n* = 67).

Variable	Overall (*n* = 67)
Demographic characteristics	
Age (years), mean ± SD	51.46 ± 21.71
Female sex, *n* (%)	35 (52.2)
Married, *n* (%)	46 (68.7)
Unemployed, *n* (%)	44 (65.7)
Burn characteristics	
TBSA (%), mean ± SD	15.48 ± 20.45
Burn depth, *n* (%)	
Superficial partial‐thickness	24 (35.8)
Deep partial‐thickness	29 (43.3)
Full‐thickness	14 (20.9)
Clinical course	
Length of hospital stay (days), mean ± SD	3.54 ± 5.15
Length of hospital stay (days), median (IQR)	1 (0–5)
Number of operations, mean ± SD	2.59 ± 2.89
ICU admission, *n* (%)	12 (17.9)
Mechanical ventilation, *n* (%)	7 (10.4)
Discharge outcome	
Partial recovery	50 (74.6)
Death	3 (4.5) (95% CI: 0.9%–12.5%)
Other/unspecified	14 (20.9)
Full recovery	0 (0.0%)

*Note:* Data are presented as mean ± SD, median (IQR), or *n* (%). All variables were available for all 67 included patients.

Abbreviation: TBSA, total body surface area.

The median hospital length of stay was 1 day (range: 0–25 days), indicating that many patients sustained relatively minor burns and were discharged shortly after definitive treatment, whereas a smaller number required prolonged hospitalization due to greater injury severity or more complex clinical management.

Neurological disorders were categorized into three broad groups: central nervous system (CNS) disorders (*n* = 46, 68.7%), spinal cord disorders (*n* = 15, 22.4%), and peripheral nervous system/neuromuscular disorders (*n* = 6, 8.9%). Among CNS disorders, cerebrovascular accidents (*n* = 23) and neurodevelopmental disorders (*n* = 13) were the most frequently observed diagnoses. Because this was a retrospective chart review, a comprehensive diagnostic classification for each neurological condition was not consistently available in the medical records, and this is acknowledged as a study limitation.

No statistically significant differences were observed between male and female patients regarding length of hospital stay (*p* = 0.329), number of operations (*p* = 0.632), TBSA (*p* = 0.232), or age (*p* = 0.244) (Table 2).

**Table 2:** Comparison of demographic and clinical characteristics by sex (*n* = 67).

Variable	Male (*n* = 32)	Female (*n* = 35)	Test statistic (*t*)	*p* value
Age (years), mean ± SD	48.03 ± 21.71	54.59 ± 23.48	−1.176	0.244
TBSA (%), mean ± SD	18.62 ± 20.30	12.53 ± 20.69	1.207	0.232
Length of hospital stay (days), mean ± SD	3.84 ± 5.06	2.62 ± 5.05	0.985	0.329
Number of operations, mean ± SD	2.77 ± 2.77	3.42 ± 3.03	0.482	0.632

*Note:* Continuous variables were compared using the independent‐samples *t*‐test. A two‐sided *p* value < 0.05 was considered statistically significant.

Abbreviations: SD, standard deviation; TBSA, total body surface area.

Correlation analysis demonstrated a strong positive association between the number of operations and hospital length of stay (*r* = 0.796, *p* < 0.001). A moderate positive association was also observed between the number of operations and TBSA (*r *= 0.479, *p* < 0.001), whereas a weak positive association was identified between TBSA and length of hospital stay (*r* = 0.281, *p* = 0.023). Age showed no statistically significant correlation with any of the other study variables (all *p* > 0.05) (Table 3).

**Table 3:** Correlations between length of stay, number of operations, TBSA, and age.

Pair of variables	Pearson rho (ρ)	*p* value
Length of stay vs. Number of operations	0.796	<0.001
Number of operations vs. TBSA	0.479	<0.001
Length of stay vs. TBSA	0.281	0.023
Length of stay vs. Age	0.242	0.052
Number of operations vs. Age	0.207	0.099
TBSA vs. Age	−0.020	0.874

*Note:* Values represent Pearson correlation coefficient (*r*) correlation coefficients.

In the multivariable negative binomial regression analysis (Table 4), the number of operations was the only variable significantly associated with hospital length of stay (IRR = 1.16, 95% CI: 1.06–1.27, *p* = 0.001). Age (IRR = 1.00, 95% CI: 0.98–1.02, *p* = 0.674) and TBSA (IRR = 1.01, 95% CI: 0.99–1.03, *p* = 0.326) were not significantly associated with length of stay. Given the retrospective design of the study and the time‐dependent nature of the number of operations, this finding should be interpreted as a contemporaneous clinical association rather than evidence of an independent causal relationship. Accordingly, the number of operations is best regarded as a marker of treatment intensity rather than an independent determinant of prolonged hospitalization.

**Table 4:** Negative binomial regression analysis of factors associated with length of hospital stay (*n* = 67).

Variable	IRR	95% CI	*p* value
Number of operations	1.16	1.06–1.27	0.001
TBSA (%)	1.01	0.99–1.03	0.326
Age (years)	1.00	0.98–1.02	0.674

*Note:* Model fit: Log‐likelihood = −188.4; AIC = 384.8; BIC = 393.2. The model was adjusted for age, TBSA, and number of operations.

Abbreviations: CI, confidence interval; IRR, incidence rate ratio.

## 4 Discussion

This retrospective cohort study described the clinical characteristics and hospital outcomes of burn patients with pre‐existing neurological disorders and evaluated factors associated with hospital length of stay. The principal finding was that the number of operations was significantly associated with hospital length of stay, whereas age and total body surface area (TBSA) were not independently associated with the outcome in the multivariable negative binomial model. However, this finding should be interpreted with caution because the number of operations accumulates during the same hospitalization in which the length of stay is measured. Consequently, this association is susceptible to reverse causation and time‐dependent bias, as patients with longer hospitalizations have a greater opportunity to undergo additional surgical procedures, whereas patients with more severe injuries or treatment‐related complications are also more likely to require repeated operations. Therefore, the number of operations should be considered a marker of treatment intensity and clinical complexity rather than an independent determinant of prolonged hospitalization.

Pre‐existing neurological disorders may influence burn outcomes through several mechanisms. Conditions such as cerebrovascular disease, dementia, spinal cord injury, and neurodevelopmental disorders can impair mobility, cognition, communication, and participation in rehabilitation, thereby increasing the complexity of wound care and inpatient management. Previous clinical studies have demonstrated that pre‐existing medical comorbidities are associated with prolonged hospitalization and poorer outcomes among burn patients, highlighting the importance of considering underlying chronic diseases when interpreting burn outcomes [8]. Likewise, Weiss et al. reported that burn patients with pre‐existing neurological disorders experienced more complicated clinical courses and longer hospital stays than patients without neurological disease, supporting the clinical relevance of neurological comorbidity in burn care [6]. Because the present study did not include a comparison group of burn patients without neurological disorders, our findings should be interpreted as descriptive and hypothesis‐generating rather than evidence that neurological disorders independently worsen burn outcomes.

In the present study, TBSA demonstrated a moderate positive association with the number of operations and a weak positive association with hospital length of stay. These findings suggest that larger burns are more likely to require operative management, but do not completely explain the variability in hospitalization duration. Previous investigations have similarly shown that burn severity alone cannot fully account for differences in clinical outcomes, as patient‐related factors and pre‐existing comorbidities also contribute substantially to resource utilization and recovery [9].

Experimental evidence also supports a biological interaction between burn injury and the nervous system. Yang et al. demonstrated that severe burn injury increased blood–brain barrier permeability and promoted neuroinflammatory responses in a murine model, providing mechanistic evidence that burn injury may adversely affect the central nervous system. However, because these findings were obtained from an experimental animal model rather than patients with pre‐existing neurological disorders, they provide biological plausibility rather than direct clinical evidence for the present findings [10]. However, because that study was performed in animals and investigated neurological injury developing after burn trauma rather than patients with pre‐existing neurological disorders, direct comparison with the present clinical findings is limited. Instead, these experimental findings provide biological plausibility for the complex interaction between burn injury and neurological dysfunction.

Several limitations should be acknowledged. First, the retrospective design is inherently subject to documentation bias and incomplete clinical records. Second, the relatively small sample size limited statistical power and restricted the number of variables that could be included in the multivariable model. Third, the absence of a comparison group of burn patients without pre‐existing neurological disorders precludes conclusions regarding the independent effect of neurological disease on burn outcomes. Fourth, only age, TBSA, and the number of operations were included in the regression analysis because other clinically relevant variables, including burn depth, inhalation injury, infection or sepsis, neurological disease severity, and baseline functional status, were not consistently available in the medical records. Fifth, neurological disorders represented a heterogeneous group of conditions that may have different effects on recovery and hospital outcomes. Finally, because the number of operations is inherently a time‐dependent variable, the observed association with hospital length of stay should not be interpreted as evidence of a causal relationship.

Despite these limitations, this study provides one of the few clinical descriptions of burn patients with pre‐existing neurological disorders. The findings should be considered hypothesis‐generating and support the need for larger prospective studies with appropriate comparison groups, standardized neurological assessments, and more comprehensive clinical data to better characterize factors associated with hospital outcomes in this patient population.

Because transfer to another facility and discharge against medical advice could not be reliably distinguished in the retrospective records, these outcomes were grouped together as “Other/unspecified,” which represents a study limitation.

## 5 Conclusion

Among burn patients with pre‐existing neurological disorders, a greater operative burden was associated with longer hospital stay. Because the number of operations accumulates during hospitalization, this association should be interpreted as descriptive rather than causal. Owing to the retrospective design, modest sample size, and absence of a comparator group, the findings should be considered hypothesis‐generating. Larger prospective studies with appropriate comparison groups and comprehensive clinical data are required to clarify factors associated with hospital outcomes in this patient population.

## Author Contributions

**Siamak Rimaz:** conceptualization, supervision, methodology. **Hossein Khoshrang:** conceptualization, supervision. **Mohammadreza Mobayen:** methodology, writing – original draft, investigation. **Mona Alipour:** data curation, writing – original draft. **Mohaya Farzin:** conceptualization, supervision, writing – review and editing.

## Funding

The authors have nothing to report.

## Ethics Statement

This study was approved by the Ethics Committee of Guilan University of Medical Sciences (IR.GUMS.REC.1403.234). Because this retrospective study used anonymized medical records, the requirement for informed consent was waived by the Ethics Committee.

## Consent

The authors have nothing to report.

## Conflicts of Interest

The authors declare no conflicts of interest.

## Declaration of AI Use

During the preparation of this manuscript, artificial intelligence‐assisted tools were used only for language editing and drafting suggestions. No artificial intelligence tool was used for data analysis, data interpretation, or generation of scientific results. All content was critically reviewed, revised, and approved by the authors, who accept full responsibility for the final manuscript.

## Transparency Statement

The lead author (Mohaya Farzin) affirms that this manuscript is an honest, accurate, and transparent account of the study being reported; that no important aspects of the study have been omitted; and that any discrepancies from the study as planned have been explained.

## Data Availability

The datasets are not publicly available due to patient confidentiality but are available from the corresponding author on reasonable request.
